# The Healthy Primary School of the Future: A Contextual Action-Oriented Research Approach

**DOI:** 10.3390/ijerph15102243

**Published:** 2018-10-12

**Authors:** Nina H. M. Bartelink, Patricia van Assema, Maria W. J. Jansen, Hans H. C. M. Savelberg, Maartje Willeboordse, Stef P. J. Kremers

**Affiliations:** 1 Department of Health Promotion, Care and Public Health Research Institute (CAPHRI), Maastricht University, P.O. Box 616, 6200 MD Maastricht, The Netherlands; p.vanassema@maastrichtuniversity.nl; 2 Department of Health Promotion, School of Nutrition and Translational Research in Metabolism (NUTRIM), Maastricht University, P.O. Box 616, 6200 MD Maastricht, The Netherlands; s.kremers@maastrichtuniversity.nl; 3 Academic Collaborative Centre for Public Health Limburg, Public Health Services, P.O. Box 33, 6400 AA Heerlen, The Netherlands; maria.jansen@ggdzl.nl; 4 Department of Health Services Research, Care and Public Health Research Institute (CAPHRI), Maastricht University, P.O. Box 616, 6200 MD Maastricht, The Netherlands; 5 Department of Nutritional and Movement Sciences, Nutrition and Translational Research Institute Maastricht (NUTRIM), Maastricht University, P.O. Box 616, 6200 MD Maastricht, The Netherlands; hans.savelberg@maastrichtuniversity.nl; 6 Department of Family Medicine, Care and Public Health Research Institute (CAPHRI), Maastricht University, P.O. Box 616, 6200 MD Maastricht, The Netherlands; maartje.willeboordse@maastrichtuniversity.nl

**Keywords:** action research, context, complex systems, health and well-being, health-promoting school, monitoring and evaluation, primary school intervention

## Abstract

*Background:* Schools can play an important role in promoting children’s health behaviours. A Dutch initiative, ‘The Healthy Primary School of the Future’, aims to integrate health and well-being into the school system. We use a contextual action-oriented research approach (CARA) to study the implementation process. Properties of CARA are its focus on contextual differences and the use of monitoring and feedback to support and evaluate the process of change. The aim of this article is to describe the use of the approach. *Methods:* Four schools (each with 200–300 children, aged 4–12 years) were included; all located in low socio-economic status areas in the south of the Netherlands. Data collection methods include interviews, observations, questionnaires, and health and behavioural measurements. Research contributions include giving feedback and providing schools with a range of possibilities for additional changes. The contextual data we examine include schools’ health promoting elements, practices of teachers and parents, dominating organisational issues, and characteristics of the student population; process data include the presence of potential barriers to changes. *Discussion:* CARA is an adaptive research approach that generates knowledge and experiences on how to deal with health promotion in complex systems. We think this approach can set an example for research efforts in comparable initiatives.

## 1. Background

Children living in low socio-economic status (SES) communities are more likely to have unhealthy behaviours that include physical inactivity and unhealthy dietary habits, compared to those living in high-SES communities [1,2]. These unhealthy behaviours can lead to health problems such as overweight/obesity, type 2 diabetes, cardiovascular diseases, and mental health problems, which can develop even at a young age [3]. Promoting healthy behaviours at an early age may help to improve children’s health, as well as their educational achievements; both may lead to improved health in later life [4]. Schools can play an important role in this, since they reach all children and form a strong social network of teachers and children who can influence one another, and since a significant proportion of a child’s day is spent at school [5]. Moreover, school and home are both part of a child’s mesosystem: changes in the school may also influence the home environment, which could enhance the effects of school health promotion [6,7].

Despite the school’s potential to help improve children’s health, school health promotion in the Netherlands at the beginning of the 21st century was marked by relatively low priority and lack of coordination and fragmentation, and was often supply-driven [8]. Traditionally, the Dutch primary school system (for children aged 4–12 years) has been mainly driven by educational requirements determined at the national level by the Ministry of Education, Culture and Science, which focus on maths, language, reading, and world orientation [9,10]. Due to these educational requirements, the interest in, and priority given to, the implementation of health promoting (HP) changes at schools is limited, as these changes do not directly address the educational requirements [8]. As a result, HP changes are only coincidentally implemented and often lack systematic coherence and sustainability, as the changes are not embedded in the school system. Whereas educational requirements have been defined at the national level, the Ministry of Health, Welfare and Sports has delegated the responsibility for health promotion to the local authorities, leading to further fragmentation. Local health organizations have to compete for the attention of schools to make them realize the importance of health promotion and its implementation. This leads to a supply-driven approach, which can irritate schools due to the abundance of initiatives offered to them that do not really match their needs [11]. On top of all these issues, the school itself is a complex system, characterized by a large number of interacting institutional elements [12]. This means that it depends on the specific school context how suitable a change towards health promotion is [13]. It also means that each implemented change will have different effects at each school; there is always an interaction between the change and the school context [13,14,15].

This complexity of the school system, together with the contextual differences between them, the fragmentation of school health promotion, and the worrying increase in unhealthy behaviours among school children, have induced the local educational board ‘Movare’, the Regional Public Health Services (RPHS), and Maastricht University (UM), all situated in the southern part of the province of Limburg, to take action. Movare’s primary schools are in the former mining area of Parkstad, in which eight municipalities around the city of Heerlen collaborate (211 square kilometres; 250,000 inhabitants). This region is characterized by a low SES, compared to the mean for the Netherlands [16,17,18]. Health and educational issues, such as a high prevalence of overweight/obesity and high school dropout rates compared to the national average, are a persistent problem in this region, and continue to exist from generation to generation [18,19,20,21]. Therefore, the three organizations (Movare, RPHS, UM) developed the ‘Healthy Primary School of the Future’ (HPSF): a Dutch initiative which aims to sustainably integrate health and well-being within the school system [18]. HPSF intends to go beyond traditional temporary and superficial top-down solutions and to establish a co-creation movement in schools towards systematic incorporation of health and well-being. This incorporation ideally leads to sustained changes that become embedded in the DNA of the school. In other words, HPSF aims to *add-in*, instead of *adding-on*, health and well-being to the school system. The initiative builds upon the principles of the health-promoting school (HPS) framework, which aims to create a healthy school environment using a school system approach. HPS focuses not only on classroom-based health education, but also on changes in school policy and the schools’ physical and social environment [22], using bottom-up involvement of pupils, parents, teachers, and staff.

Co-creation processes within the school system are challenging for researchers to study in a scientifically sound manner. Traditionally, many action researchers in school interventions have followed the cycle of needs assessment, development, implementation, monitoring, and evaluation of change [23,24]. However, the limitation of following these steps is that they suggest a logical, causal process, which is difficult to identify in a complex school system initiative [25], where changes are interacting with each other and with other contextual aspects of the school [15,26]. Therefore, we felt the need to find a way to adapt our research to this complexity. As this study is not the first to deal with initiatives in complex systems, we have been inspired by the existing literature, from which various considerations and insights were gathered [12,13,26,27,28]. These insights into systems thinking have led us to adapt the principles of action research into a contextual action-oriented research approach (CARA). The purpose of CARA is to contribute as researchers to the process of a complex intervention initiative, and to conduct a thorough evaluation of the process and its final outcomes that addresses the importance of the implementation context. Basic properties of CARA are its specific focus on contextual differences, and the use of monitoring and feedback to both support and evaluate the process of change. The approach centers around four key questions: *(1) What is the pre-existing context of each school?; (2) How does the process of change in each school evolve and which factors affect this process?; (3) How can research contribute to the process of change?;* and *(4) Do children’s health and health behaviours improve as a result of the HP changes?*

The aim of this article is to elaborate on the way in which we are dealing with the complexity of the school system and the HPSF initiative by using CARA and how we are able to contribute to the initiative and at the same time conduct a thorough evaluation. HPSF has a broad focus on different aspects of health and well-being. The present study focuses on two key aspects, i.e., healthy nutrition and physical activity (PA).

## 2. Methods

### 2.1. The Healthy Primary School of the Future

Four schools, each with 200–300 children (aged 4–12 years) and with 15–30 teachers per school, are included in the HPSF initiative (whose implementation started in November 2015) as pilot schools. In addition, four comparable control schools in the same region were also included. Information about the recruitment of these eight schools has been described by Willeboordse et al. [18]. The three cooperating organizations, Movare, RPHS, and UM, have introduced two top-down changes to the schools’ system: (1) providing a free healthy lunch each day; and (2) a full hour of structured PA each day, both prepared and led by external pedagogical staff, provided by childcare organisations [18]. While in other national school systems these may represent usual practice, these changes are hypothesized as disruptive to the Dutch school system, because the provision of school lunches and structured PA sessions are not usual practice in Dutch schools. The schools involved teachers and parents in the one-year decision and development process to adapt the two changes to their context. The schools only decided to start implementation of the two changes if they had the teachers’ support and at least 80% parental support. It is expected that the changes will create increased interest of the school in healthy nutrition and PA. In addition, it is assumed that they will create momentum for additional HP changes fitting the context of each school. As part of the HPSF initiative, one teacher in each school is appointed as school coordinator. She or he develops each change with working groups of parents, teachers, and children, as well as two closely involved employees of the RPHS (a youth nurse and a health promoter) who are assigned to each school to provide support when needed. In addition, a project team was created, including the schools, Movare school board, UM, child care organizations, catering services, sports and leisure organizations, RPHS, and the Limburg provincial authorities. The provincial authorities will continue to support the project financially until 2019 to realize a breakthrough in the worrying health status of the young generation.

### 2.2. Contextual Action-Oriented Research Approach (CARA)

The aim of research into the HPSF initiative is not only to evaluate, but also to support the process of change in the schools, with a specific focus on the contextual differences. To be able to achieve this, we used CARA, which builds on our previous experiences in school health promotion and on the international literature regarding new insights into complex systems thinking [12,13,14,15]. CARA is an adaptation of action research principles, whereby the traditional linear steps are let go as they suggest a logical, causal process. In contrast, CARA aims to identify where changes are interacting with contextual aspects of the school [15,26]. Table 1 shows how the traditional steps of action research (column 1) are combined with the insights of complex systems thinking (column 2) to form CARA. The table also presents the methods, based on the four key questions (column 3). For each key question, the relevant insights and methods are discussed in more detail below.

#### 2.2.1. What Is the Pre-Existing Context of Each School?

This first key question regards examining the school context to determine the starting point of HPSF. Each school context is part of a complex system [12]. Therefore, introducing fundamental changes to a system first requires an understanding of this context [28]. Thus, the first phase involves the investigation of each school context. Each school has decided autonomously whether to participate in HPSF. Existing literature shows that in this decision and the further process of change, several contextual factors might be of direct importance, e.g., HP practices of teachers and parents; HP elements in the school (school routine, policy, education, and environment); dominating organisational issues (e.g., staff turnover); innovation-, implementers-, organisation-, and socio-political context-related barriers for HPSF perceived by implementers; and characteristics of the student population (health, well-being, health behaviours, demographics) [29,31]. To assess the school context, we use mixed methods that are appropriate to obtain rich information, and that can rapidly be translated into real-time feedback for the schools. For all methods, we use a framework of possible behavioural goals regarding healthy nutrition and PA (Table 2). These goals were defined by the research team during the preparation year, by applying insights from the Precede-Proceed model about ways to define clear behavioural goals [36]. In addition to their use as a framework for the researchers, the goals may also work as an inspiration for the schools to define their needs and preferences to focus on specific aspects.

*Interviews:* Semi-structured interviews are conducted with the school coordinator and the health promoter in each school. The behavioural goals are used as topics in these interviews. The aim of the interviews is to draw up an overview of the HP elements in the school and a broad understanding of any dominating organisational issues in the school. The results are summarized in an overview, checked by the interviewees, and fed back to the project team.

*Barrier questionnaire:* To examine which factors in the context are perceived to be potential barriers for the process of change, all teachers and external pedagogical staff are asked to complete a questionnaire. The used questionnaire was based on the Measurement Instrument for Determinants of Innovations (MIDI), a Dutch validated questionnaire developed by Fleuren et al. [31] and used in several Dutch studies [37,38]. The questionnaire contains 46 statements, related to innovation-, implementers-, organisation-, and socio-political context-related barriers for HPSF affecting innovation adoption, implementation, and integration. Responses to each statement range from 1 (totally disagree) to 10 (totally agree). Statements with an average score below 6 are defined as potential barriers. This corresponds to the grading system used in Dutch primary schools, which also uses a range from 1 to 10 for school tests, and scores below 6 as insufficient or fail. 

*Practices questionnaire:* A questionnaire, based on previous work by Gevers et al. [39] and O’Connor et al. [40], is used to examine HP practices of teachers at school and parents at home, e.g., rules, modelling behaviour, encouragement, and availability. The existing questionnaires were used in several previous Dutch studies [41,42]. The items in the questionnaire focus on parents in the home setting. Therefore, we rephrased the items in the teacher questionnaire to the school setting. To deal with validity and reliability concerns, we pre-tested the instruments, and we will check for variability per question and will calculate Cronbach’s Alpha afterwards. A Likert-scale from 1 (completely disagree) to 5 (completely agree) is used for the answers.

*Health and behavioural measures*: Measurements of children’s health and health behaviours are carried out by researchers during one week at the beginning of the school year [18]. Inter-rater variability was minimised by training the researchers according to a strict protocol. The children wear an accelerometer for the whole week to objectively assess their PA levels. Their height, weight, and waist and hip circumference are measured during the physical education lessons. Children’s dietary and PA behaviours are assessed using a questionnaire for the children during class hours and a digital questionnaire for their parents. Ethical approval (14-N-142) was given by the Medical Ethics Committee Zuyderland located in Heerlen (Parkstad, the Netherlands). Parents had to sign an informed consent to participate in all measurements for themselves and their child(ren). More detailed information about these measurements has been published by Willeboordse et al. [18].

#### 2.2.2. How Does the Process of Change in Each School Evolve and Which Factors Affect This Process?

The second key question aims to examine the process of change, i.e., the adoption, implementation, and integration of the HP changes, and its interaction with the school context. Since HPSF takes place in a complex system, some aspects are important to take into consideration. First, the process of change in each school may not be characterized by a linear cause-effect relationship. This non-linearity means that small changes can produce large effects at a so-called ‘tipping’ point [12,30]. When this tipping point is reached is hard to predict. Therefore, both the innovators in the school and all external experts involved need to be receptive to what emerges and expect the unexpected. Second, a complex system means adaptation: interacting elements and people in an environment respond and adapt to each other [12]. This means that what emerges in a school is interpreted as a function of on-going adaptations that may continually lead to new needs, interests, and opportunities in the school. In this context, Reiser et al. [33] stated that the implementation of a change is more successful and leads to greater ownership and commitment if it involves a process of mutual adaptation. This indicates a bidirectional process in which a proposed change is modified to suit the needs, interests, and opportunities of the school, and in which the people at the school are open to (major) adjustments and adjust to meet the requirements of that change. In essence, this requires a combined top-down/bottom-up process, which is another aspect to take into consideration when changing a complex system. A bottom-up approach is needed as teachers, children, and their parents know best which changes are most appropriate to their school. Hence, they should be the ones to lead the process of change. A top-down approach is needed as the external experts involved have specific health promotion knowledge, skills, and experiences, which may lead to more effective changes [30,34]. It is important not only to find a balance between these two, but also to be constantly aware of the primary source of the idea for a change as well. When an idea develops bottom-up, the external experts should help the school by using their specific knowledge and experiences. When the primary idea is introduced top-down, the external experts should help the school to encourage involvement among the people in the school and help them make contextual adjustments to fit the proposed change to the system.

To facilitate this on-going process of change in each school, we evaluate the process by continuously monitoring the changes and their consequences. The results have to be concrete and specific, so useful feedback and recommendations can be given to the schools to guide their actions and help them adapt to the changes. In the course of the study, all monitoring results are combined to create more abstract and general recommendations to help other people or organizations who want to start HP initiatives in schools. The process of change is monitored using mixed methods.

*Interviews:* Annual interviews are conducted with the school coordinator and the health promoter of each school to discuss the HP changes in the school, their development and implementation, and the influencing factors associated with them.

*Observations:* A researcher participates, observes, and takes notes in all meetings of the project team and meetings of the health promoters. This researcher also conducts observations in the four schools, with the aim of learning about the school’s dynamics and to see and hear influencing factors of HPSF (rather than as a form of fidelity assessment). To create openness, no observational checklist is used. During these school visits, the researcher randomly talks to staff and children in the school to hear about their experiences and perceptions regarding HPSF. Observations will take at least one full week each year during effect measurements of and frequent visits (at least once every three months) to each school during the year. Notes will be taken during and immediately after visiting the school. All observational notes provide qualitative data about HPSF and any experienced influencing factors.

*Barrier questionnaire:* It is expected that different barriers will appear during different phases in the process of change. Therefore, twice a year, all teachers and external pedagogical staff are asked to complete the same questionnaire as described above to address the first key question. Statements with an average score below 6 are defined as possible barriers. Some open questions have been added to obtain the respondents’ opinions on how the process is going.

*Practices questionnaire:* The process of change can also have an impact on the practices of teachers and parents. Therefore, the same questionnaire as described above to address the first key question is annually filled in by the teachers and parents. 

#### 2.2.3. How Can Research Contribute to the Process of Change?

The third key question is intended to examine supportive contributions of research to the process of change at the schools. This is achieved by embedding the research in the HPSF initiative, similar to its role in action research [35]. As a result, evaluation is no longer merely an external observation of strategies to implement changes, but becomes one of the strategies itself [12]. The attitude of the researchers in this approach is also different: they are no longer neutral and fully objective, but join in the discussions and give support to the innovators whenever possible on the basis of their professional knowledge, skills, and experiences, as well as the results of the monitoring data [12]. It is expected that regular feedback will provide valuable guidance to the process of change in the schools [35]. Examples of feedback from the researchers to each school are written summaries of the most important results of the interviews; overviews of the perceived barriers for the teachers and external pedagogical staff; and short, easily understandable animated videos of the most important results of the health and behavioural measures. Furthermore, as part of the feedback, we aim to include suggestions for focal points to further improve HPSF. Other contributions of the researchers on top of the feedback they provide are the suggestions for possible behavioural goals and the offer of a selection of relevant, previously developed, and evidence-based additional changes for the schools. To be able to offer this as a range of possibilities, a so-called ‘*fruit basket model*’ is introduced. This ‘basket’ consists of a continuously expanding overview of available evidence-based additional changes (‘fruits’) that schools can introduce. Examples of such changes are gardening activities, energizers in the lessons, and creating a PA-friendly schoolyard. According to the school’s needs, interests, and opportunities, the school coordinator decides together with the working groups what the school’s focus will be, and which specific ‘piece of fruit’—additional change—fits their school. This change is then adapted to the specific school context, with the help of external experts, before implementation starts. An overview of the current ‘fruit basket’ is presented as Table S1. Questions regarding the researchers’ contributions are included in the interviews to evaluate the extent to which the contributions are experienced as supportive, and/or whether other contributions are desirable.

#### 2.2.4. Do Children’s Health and Health Behaviours Improve as a Result of the HP Changes?

The fourth key question is intended to determine the influence of HPSF on children’s health and health behaviours to examine how, for whom, and in what circumstances the initiative works. To examine if HPSF leads to changes in children’s health and health behaviours, an effect study is being carried out with a quasi-experimental design [18]. Data on children’s health and health behaviours are gathered during annual measurement weeks between 2015 and 2019 at all four HPSF schools and at four control schools (approximately 1700 children, 900 parents, and almost 80 employees). However, changes may have different effects in different contexts, even if their implementation does not vary [13]. Therefore, to investigate for whom and under which circumstances the changes have the greatest effect, we examine the differences in effect between the schools by combining the results of the effect evaluation with relevant implementation and contextual factors of the schools. The implementation and contextual variables which are collected quantitatively at both the intervention and control schools, e.g., characteristics of the student population and HP practices of teachers and parents, are included as potential moderators in the analyses. Moderator analyses have been defined as a fundamental step in understanding behaviour change and are conducted by using an interaction term in the statistical models and (in the case of significant interactions), stratifying the data by a moderator to re-examine the effect [43]. The quantitative implementation and contextual data that are only collected in the four intervention schools, e.g., perceived barriers as measured by the questionnaire, are analysed on changes over time and compared between the schools. Finally, qualitative implementation and contextual data that are collected in the four intervention schools, e.g., school specific influencing factors on the process of change, are used in a comparative manner between the schools to provide additional insight or so-called illumination on the process of change [44].

## 3. Discussion

The present paper has introduced the contextual action-oriented research approach that we developed to deal with the complexity of both the school system and the HPSF initiative. The paper has shown how we aim to contribute to the initiative and at the same time conduct a thorough evaluation. Some methodological, practical, and/or integrity limitations and strengths of the approach need to be discussed. First, fully assessing and understanding all aspects of each context, the process of change, and the implementation of each change, is impossible due to limitations of time, resources, and participant burden [13]. Therefore, CARA researchers have to make difficult selection choices about which data to collect and in how much detail. Luckily, we do not have to start from scratch. We build on previous work published in the international literature on, e.g., relevant concepts in the process of change in complex (school) systems, which was essential for this decision process. We included methods that are appropriate to obtain rich information, that are feasible for the researchers and the schools, and that can be translated into rapid and real-time feedback for the schools.

Second, we believe that the data collection instruments in CARA need to fit the context to be able to get meaningful data. Therefore, existing instruments might not be available or have to be adapted to the context. In the current study, we have adopted the strategy of preferring adjustment of existing and tested instruments over the development of new instruments. By using the principle of data triangulation in our analysis, we combine the accuracy of the quantitative questionnaires with the in-depth insights that interviews and observations afford.

Third, CARA includes not only an evaluation of the process of change in the schools, but also an effect study to investigate the evidence for the behavioural and health effects of these changes among the children. Since randomization is neither desirable nor feasible here [26], a quasi-experimental study design is used. To investigate for whom and under which circumstances the changes cause the greatest effect, the school-specific effects are combined with relevant process and contextual factors at each school. Although we do recognize the importance of assessing the implementation fidelity, the focus in our study is not on the fidelity of intervention components, but on identifying when and how adaptation take place, and which factors prove to be crucial for sustained changes. This specific focus of interest is based on the notion that even small changes may produce large effects in a specific context (i.e., ‘tipping’ point) [12,30]. Better (i.e., high fidelity) implementation of a change does thereby not necessarily mean greater effect [13]. Another aspect to consider is that CARA researchers are not external observers, but actively participating partners in the initiative. By including an effect study with a quasi-experimental design in CARA as well, we believe we combine the best of two worlds: the advantages of a researcher involved in the process of change and still being able to study the effects objectively.

Finally, CARA also implies time-consuming research, as a thorough insight into the school context is necessary. This insight requires a relationship of trust between the researchers, the schools, and all other partners involved, which takes time to build. In this relationship, nobody should be afraid to say what really bothers them, which should yield data that reflect the real situation. Moreover, CARA requires flexible time planning of the researchers; they need to be able to react quickly to what happens in each school, to be able to give relevant support, and to analyse the data quickly to be able to give rapid and real-time feedback. At the same time, the feedback process needs to take place in a careful manner, as both the initiative and the research can benefit from an open discussion of the real situation of those involved without losing the trust of the informants. To maintain a relationship of trust, we aim to offer honest but discrete feedback. When results cannot be fed back anonymously, we aim to ask permission of the informants before communicating the feedback to others. CARA can also be time-consuming for the schools. Researchers need to consider this and ensure that studying the initiative is feasible for them and the schools. Due to our critical selection of mixed methods, we believe we have found a feasible way to support and evaluate the initiative in the schools. By means of the feedback provided and the focal points included to further improve HPSF, we aim to offer added value to the schools which we hope outweighs their time investment.

In addition to these methodological, practical, and integrity aspects, there is another important aspect to consider: whereas the current paper mainly focuses on the school complexity, the school is only one of a child’s microsystems. Changes in children’s home setting and neighbourhood, their other microsystems, also interact with the impact of changes at school [7]. Thus, the complexity goes beyond the focus of the research described in this paper.

## 4. Conclusions

Overall, we think that CARA is a possible solution to the challenge of supporting and evaluating change in school-based initiatives. CARA generates knowledge and experiences on how to deal with health promotion in complex systems. This paper shows an innovative approach to contribute to the process of a complex intervention, including a thorough evaluation of the process and its final outcomes that addresses the importance of the implementation context. We think that CARA can be an example for research efforts in comparable initiatives and can help to make sustainable (add-in) changes in complex systems.

## Figures and Tables

**Table 1:**
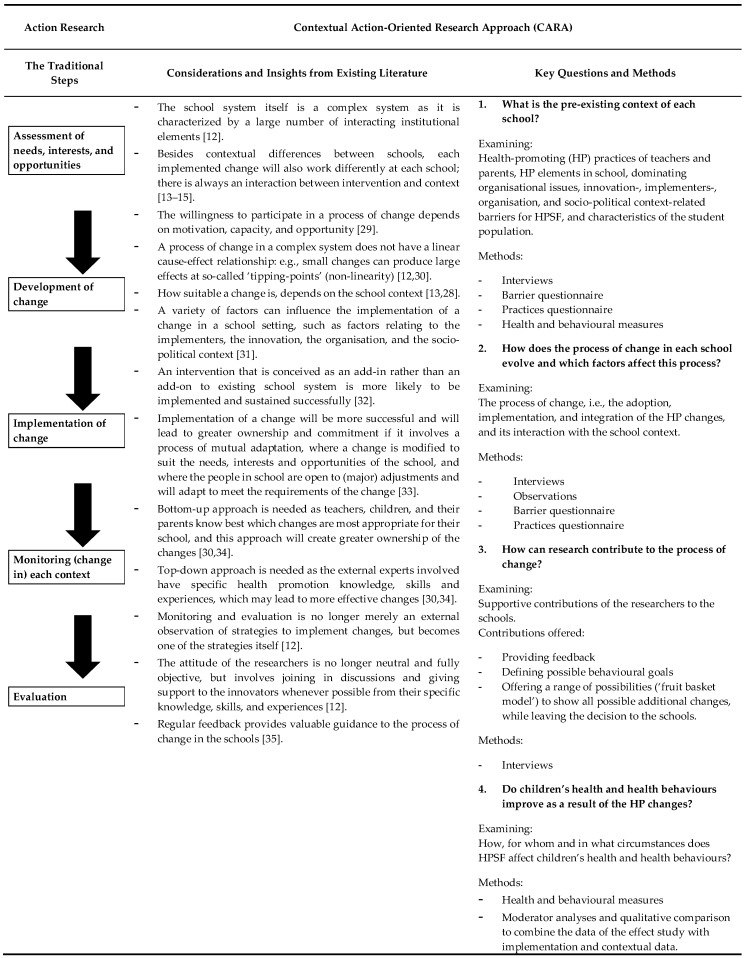
Considerations and insights on initiatives in complex systems.

**Table 2:** Behavioural goals regarding physical activity and healthy nutrition.

Physical Activity	Healthy Nutrition
- Children engage in moderate to vigorous physical activity (MVPA) for at least 60 min/day.	- Children consume a healthy breakfast every day.
- Children use active school transport (cycling, walking) if the distance to their school is less than 2 km, or children are taken to school by active transport.	- Children consume a healthy lunch every day.
- Children are physically active (MVPA) for at least 20 min/day during school breaks.	- Children consume two (different) pieces of fruit a day.
- Children are not sedentary for more than 30 consecutive minutes. Every 30 min, children should have a 2-min break involving walking, standing or moving.	- Children consume 100–200 g of vegetables a day.
- Children are physically active (MVPA) for at least 20 min/day during physical education lessons (lasting 1 h) at least three times a week.	- Children replace energy-dense snacks with healthier alternatives.
- Children do not have more than 2 h/day of sedentary screen time (television/computer/tablet).	- Children drink water instead of sugar-sweetened drinks.
- Children take part in afterschool physical activities (e.g., sports clubs, afterschool physical activity programmes, and free time outdoor play).	- Children do not drink sports or energy drinks.
- Children are physically active (MVPA) for at least 60 min/day during the weekend.	

## Supplementary Materials

The following are available online at http://www.mdpi.com/1660-4601/15/10/2243/s1, Table S1: Overview of the current ‘fruit basket’.
