# Supplementary material for: The Healthy Primary School of the Future: A Contextual Action-Oriented Research Approach
Source: Int J Environ Res Public Health. 2018 Oct 12;15(10):2243. doi: 10.3390/ijerph15102243 (PMC6209969; doi:10.3390/ijerph15102243)
Supplement: Supplementary file 1 [file ijerph-15-02243-s001.pdf]

Additional file 1: Overview of the current 'fruit basket'

<table>
<tr>
<td><u>Aim</u>

Integrating health and well-being within the school system</td>
<td><u>General tips:</u>
Always keep the fun-factor alive.
Involve school team, parents, and children in developing and implementing changes and assessing the needs, interests and opportunities by forming working groups and student councils.
Make use of the knowledge and experience of the partners in the project, such as employees of the Public Health Service.
Share good ideas and experiences.
Talk with children about health and well-being.
Set a good example for the children.
Evaluate regularly.</td>
</tr>
</table>

<table>
<tr>
<th>School aspect</th>
<th>Possible changes</th>
<th>Brief explanation</th>
<th>Websites (for more information and/or examples)</th>
<th>Tips and other useful information</th>
</tr>
<tr>
<td rowspan="2">Policy</td>
<td>Healthy school policy</td>
<td>Changing existing policy into healthy lifestyle stimulating policy.</td>
<td>https://www.gezondeschool.nl/primair-onderwijs/toolkit-gezonde-school</td>
<td rowspan="2">- Possible issues for policy: lunch, snacks, drinks, energizers, and teaching about nutrition and exercise.
- Collaborate with the child partners for comparable policies at pre-school and after-school care.</td>
</tr>
<tr>
<td>Celebration policy</td>
<td>Developing a school policy on treats to celebrate the birthdays of children and teachers as a school.</td>
<td>http://www.voedingscentrum.nl/Assets/Uploads/voedingscentrum/Documents/Consumenten/Mijn%20kind%20en%20ik/Naar%20school/Traktatieboekje.pdf</td>
</tr>
<tr>
<td rowspan="2">Physical environment</td>
<td>Physical activity friendly schoolyard</td>
<td>Changing the schoolyard to create a physical activity friendly environment.</td>
<td>http://beheer.nisb.nl/cogito/modules/uploads/docs/64041399972677.pdf</td>
<td>- Mark the schoolyard with lines.
- Teach children fun PA games to do in the schoolyard.
- Make a schedule so that classes do not use the yard simultaneously.
- Create for each class a materials box that fits the age and interests of the group.</td>
</tr>
<tr>
<td>Water taps in school</td>
<td>Installing water taps in the school where children can refill their water bottles.</td>
<td>https://kraanwatergraag.nl/school/watertappunt/

https://www.ggdzl.nl/professionals/diensten/advisering/drankenbord-weet-jij-wat-je-drinkt/</td>
<td>- Give children a personal water bottle so that water drinking becomes a habit.
- A poster on drinks (with supporting educational lessons) can be obtained from the RPHS. It shows how much sugar is contained in the most popular drinks and how many minutes of exercise they need to burn it.</td>
</tr>
</table>

	**School garden**	Create a school garden: children can learn and experience where food comes from and become more aware of nutrition.	www.schooltuinproject.nl	- Materials and educational lessons are available from the national school garden project.
**Social environment**	***'Taakspel'* programma** (Task game programme)	A group-based approach in which children learn to comply with rules in the classroom.	http://www.taakspel.nl/	- The Task Game has been developed for grades 3 to 8. There is also Task Play in the playground, which enables primary schools to introduce rules for the playground.
**School aspect**	**Possible changes**	**Brief explanation**	**Websites (for more information and/or examples)**	**Tips and other useful information**
**Education**	***'LekkerFit'* programma** (Enjoy being fit programme)	Healthy lifestyle education with physical activity and nutrition lessons as part of the curriculum. For all school grades.	www.lekkerfitopschool.nl	
	***'Op voeten en fietsen'* programma** (By foot and by bike programme)	Inter-class competition focusing on active transport.	http://vvn.nl/wat-we-doen/op-de-basisschool/	- Investigate if a safe school route can be defined and whether the bicycle storage is large enough.<br>- Talk to the municipal authorities about the traffic rules around the school.
	***'Smaaklessen'* programme** (Taste lessons programme)	Practical food tasting lessons. For all school grades.	www.smaaklessen.nl	- Fresh products must be purchased for each lesson.<br>- Lessons can be downloaded free of charge.
**Classroom-based**	**Energizers**	Introducing short breaks of physical activity during or between lessons.	https://www.101werkvormen.nl/tag/energizer/<br><br>http://hopsports.com/what-is-brain-breaks	- Wide range of books and PA cards can be purchased for inspiration and tips. In addition, many examples are available on the internet.<br>- Make clear arrangements with the class about the energizers (indicating when the energizer ends).<br>- The PA cards can also be used during breaks or taken home.
	**Daily Mile**	Walk or run a mile each day with	http://thedailymile.nl	- Just give it a try!

		the class.		- Establish a clearly defined route for The Daily Mile.
**Physical education (PE)**	**Increase quantity**	At least 2 to 3 one-hour PE lessons a week.		- Ask how other schools schedule the 2 to 3 PE classes in a week.
	**Increase quality**	Increasing the intensity of PE lessons by using tips by Slingerland et al (32).		
	**Swimming lessons**	Introducing swimming lessons.		
**Other PA stimulating changes**	**Sport clubs**	Introducing lessons by sports clubs.	https://schoolsportvereniging.nl/index.html	- Connect with organizations/ people/sports clubs in the neighbourhood.<br>- Also check the possibility of employing trainee sports teachers to guide the PA activities.
	**Physical activities after school**	Creating PA activities after school.		
	**Natuursprong** (Nature jump)	Programme giving children the opportunity to play and be physically active in nature.	https://www.huisvoorbeweging.nl/bewegen-stimuleren/natuursprong/over-natuursprong/	
**Other healthy nutrition stimulating changes**	**Kokkerelli** (kids university for cooking)	Children (grades 5-8) visit a farm to experience and learn about food production.	http://www.kokkerelli.nl/kinderen/wie-zijn-we/	
	**SuperChefs**	Children (grades 7 and 8) learn to prepare healthy meals.	www.voedingscentrum.nl/superchefs	
	**SuperShopper**	Two lessons and a trip to a supermarket to learn about and experience making healthy choices.	http://www.voedingscentrum.nl/professionals/gezond-op-school/basisonderwijs/supershopper.aspx	
**School aspect**	**Possible changes**	**Brief explanation**	**Websites (for more information and/or examples)**	**Tips and other useful information**
**Parents**	**General tips:**<br>• Personalize: parents may have some knowledge or expertise they would like to share or use with the children.<br>• Let children and their parents do things together.<br>• Combine a parent meeting with something that involves their child, such as a play by the children or an exhibition of the children's creative works.<br>• Set the children a homework assignment (on healthy behaviour) that they should do together with their parents.			
	**Goedkoop gezonde voeding** (good food programme)	This programme teaches adults to eat healthily even on a low budget.	http://goedkoopgezondevoeding.nl	

	**E- learning on healthy lifestyles**	A free online course to give parents tips and tricks on how they can encourage their child to eat healthier food and become more physically active.	www.gezondeafsprakenmetjekind.nl	
	**Interactive theatre *'Voor je het weet, zijn ze groot'*** (They are grown up before you know it)	An interactive theatre event providing parents with information and examples and discussing raising children, healthy nutrition and PA.	http://www.voedingscentrum.nl/interactief-theater	
	**Triple P meeting**	A meeting to talk about and learn practical skills regarding difficulties of raising a child in a healthy way.	http://www.triplep-nederland.nl/nl-nl/de-triple-p-methode/triple-p-toolbox/informatie-en-lezingen-niveau-2/	
	**Triple P online**	Eight online sessions to learn strategies for raising a child in a healthy way.	http://www.triplep-nederland.nl/nl-nl/de-triple-p-methode/triple-p-toolbox/triple-p-online/	
